# *Announcement*: World Pneumonia Day — November 12, 2017

**DOI:** 10.15585/mmwr.mm6644a7

**Published:** 2017-11-10

World Pneumonia Day, observed on November 12 each year, aims to highlight the huge toll pneumonia takes on children and adults worldwide. In 2015, an estimated 2.7 million persons died from respiratory infections, including 700,000–920,000 children aged <5 years (*1*,*2*). *Streptococcus pneumoniae* and *Haemophilus influenzae* type b bacteria cause most of these deaths; however, viruses, including influenza and respiratory syncytial virus, also have a considerable impact (*1*).

Multiple vaccines are available to help prevent pneumonia, including *Haemophilus influenzae* type b, influenza, measles, pertussis, pneumococcal, and varicella vaccines. Expanding the use of the pneumococcal conjugate vaccine in childhood immunization programs around the world has reduced disease incidence in recent years, particularly among children aged <5 years (*3*). Despite progress globally in reducing the incidence of pneumonia, recent U.S. outbreaks serve as a reminder of the importance of maintaining high vaccination coverage to prevent pneumonia. For example, as of August 25, 2017, more than a quarter (28%) of 79 measles patients in a Minnesota community with low measles-mumps-rubella vaccination coverage required hospitalization, primarily for treatment of dehydration or pneumonia (*4*).

In addition to vaccination, other strategies have been proven to help prevent pneumonia. Adherence to antibiotic use guidelines reduces the development of antibiotic resistance among pneumonia-causing organisms. In addition, access to tobacco cessation programs (*5*), decreased exposure to secondhand smoke (*5*) and reduction in indoor air pollution from biomass smoke in developing countries (*6*) are important pneumonia prevention strategies that can save lives. Continued efforts to improve access to appropriate treatment for those who get pneumonia are also needed. Information about World Pneumonia Day, including the 2017 Pneumonia and Diarrhea Progress Report, is available at http://stoppneumonia.org/.
